# Causal effects of Parkinson’s disease on the risk of osteoporosis: A two-sample Mendelian randomization study

**DOI:** 10.1097/MD.0000000000040061

**Published:** 2024-11-08

**Authors:** Yu Huang, Nan Yi, Qinglong Li, Song Guo, Bingfeng Mo, Dong Yin, Hongmian Li

**Affiliations:** a The First Affiliated Hospital of Jinan University, Guangzhou, P. R. China; b Department of Orthopedics, The People’s Hospital of Guangxi Zhuang Autonomous Region & Guangxi Academy of Medical Sciences, Nanning, Guangxi, P. R. China; c Department of Gastroenterology, The People’s Hospital of Guangxi Zhuang Autonomous Region & Guangxi Academy of Medical Sciences, Nanning, Guangxi, P. R. China; d Jinan University, Guangzhou, P. R. China; e Research Center of Medical Sciences, The People’s Hospital of Guangxi Zhuang Autonomous Region & Guangxi Academy of Medical Sciences, Nanning, China.

**Keywords:** bone mineral density, genetic variants, Mendelian randomization, osteoporosis, Parkinson disease

## Abstract

Employing a two-sample Mendelian randomization (MR) analysis, we aimed to investigate the potential causal effect of Parkinson disease (PD) on osteoporosis. We conducted an in-depth MR analysis by leveraging extensive genome-wide association study datasets from the International Parkinson Disease Genomics Consortium and the Genetic Factors for Osteoporosis Consortium. We meticulously selected instrumental variables based on strict criteria, including significance thresholds, linkage disequilibrium, and the exclusion of confounding single-nucleotide polymorphisms. Our investigation utilized diverse MR methods, including inverse variance weighted, MR Egger regression, weighted median, and MR-PRESSO, to robustly evaluate the causal relationship. Our comprehensive analysis revealed noteworthy associations between PD and distinct measures of bone mineral density (BMD) (forearm BMD, femoral neck BMD, lumbar spine BMD). Specifically, the inverse variance weighted method underscored potential significant relationships between PD and forearm BMD (*P* = .037; odds ratio [OR], 1.04; 95% confidence interval [CI], 1.00–1.09), femoral neck BMD (*P* = .034; OR, 1.02; 95% CI, 1.00–1.05), and lumbar spine BMD (*P* = .043; OR, 1.03; 95% CI, 1.00–1.06). The consistency of results across various methods and sensitivity analyses indicated both robustness and minimal pleiotropy concerns. Through a two-sample MR approach, this study establishes a plausible causal relationship between PD and decreased BMD. The outcomes underscore the urgency of targeted interventions to mitigate bone loss and manage osteoporosis in individuals with PD.

## 1. Introduction

Parkinson disease (PD) is a progressive neurodegenerative disorder marked by the degeneration of dopaminergic neurons within the substantia nigra, leading to motor disturbances, including tremors, rigidity, and bradykinesia.^[1]^ Definite PD diagnosis requires the presence of at least 2 of 3 cardinal signs (akinesia, rigidity, and tremor), the absence of exclusion criteria (ophthalmoplegia, pyramidal or cerebellar signs, early dementia, urinary incontinence, postural instability, and prior exposure to neuroleptic drugs), and a positive and sustained response to levodopa therapy.^[2]^ Most PD cases (>80%) meet the criteria for definite PD diagnosis.^[2]^ With its prevalence projected to double over the next 3 decades, PD ranks as the second most prevalent neurodegenerative condition.^[3]^ Recently, the global landscape of neurological disorders has witnessed the rapid ascent of PD, a trend associated with a twofold surge in its global burden over the previous generation.^[4–7]^ While PD research has traditionally centered on its neurological implications, a growing body of evidence has begun to illuminate potential intersections between PD and broader systemic conditions.

Osteoporosis is a prevalent metabolic bone disorder characterized by diminished bone mineral density (BMD) and perturbations in bone microarchitecture, increasing the risk of fractures.^[8–10]^ In patients with PD, osteoporosis significantly increases morbidity by elevating the risk of falls and fractures. This confluence of PD and osteoporosis adds complexity to this context. Previous studies have indicated an elevated fracture risk among patients with PD, often accompanied by a notable prevalence of osteoporosis.^[11]^ However, while some findings have suggested that PD was the strongest single predictor of fractures,^[12]^ this association was not independent of BMD. There seems to be no interaction between PD and osteoporosis concerning the occurrence of fractures, indicating no effect modification by osteoporosis.^[13]^

Several studies have investigated the link between PD and BMD. However, these studies often overlooked genetic confounders, an oversight, which this study addresses through Mendelian randomization (MR). To rigorously interrogate the potential causal underpinnings of the interplay between PD and osteoporosis risk, we have undertaken an MR study. MR, a robust analytical framework, harnesses genetic variants as instrumental variables to establish causal links between an exposure (PD) and an outcome (BMD), grounded in the principles of randomization.^[14]^ The inherent randomness in the inheritance of genetic variants during conception mitigates susceptibility to confounding and reverse causation biases, rendering MR a compelling avenue for unraveling causal relationships in observational data.^[15]^ Through this study, we endeavor to provide deeper insights into the intricate relationship between PD and the vulnerability to osteoporosis, shedding light on potential avenues for targeted interventions and improved patient care.

## 2. Methods

**Figure 1.**
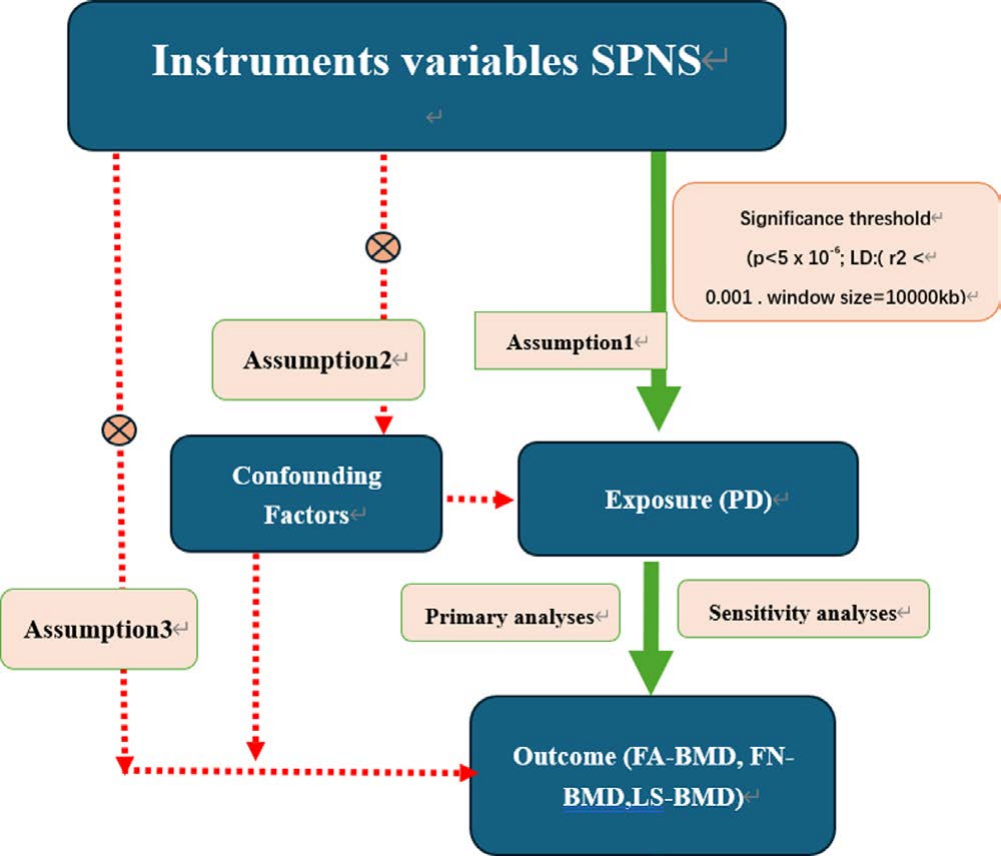
An overview of the study design. Assumption 1: the instrumental variables (IVs) must be robustly associated with the exposure. Assumption 2: the IVs must not be associated with any confounding factors of the exposure–outcome relationship. Assumption 3: the IVs must only affect the outcome through the exposure. *Primary analyses: inverse variance weighted (IVW) method, weighted median estimator, and MR Egger regression. ^†^Sensitivity analyses: MR-PRESSO, heterogeneity, Cochran Q test, and leave-one-out analyses. FA-BMD = forearm bone mineral density; FN-BMD = femoral neck bone mineral density; LS-BMD = lumbar spine bone mineral density; SNP = single-nucleotide polymorphisms.

Data sources for exposure and outcome were obtained from genome-wide association study (GWAS) data. The inclusion criteria for selecting GWAS data encompassed published, quality-assured GWAS datasets, ensuring the most recent data availability and completeness. The exclusion criteria were as follows: unpublished GWAS data, older datasets, data containing confounding factors, and incomplete data. The study design is presented in Figure 1.

### 2.1. Data sources for exposure

To obtain a more comprehensive and reliable conclusion regarding the causal link between PD and BMDs, we selected the largest GWAS published to date from the International Parkinson Disease Genomics Consortium. The European cohort is the largest dataset available to the research team, and the choice of a European cohort was made considering the accessibility of the data and the practical feasibility of the study.

The analysis was a fixed-effects meta-analysis across 17 datasets, all of which underwent a similar process of quality control for inclusion.^[16]^ The data included information from 33,674 patients diagnosed with PD and 449,056 controls from European ancestry samples. The number of single-nucleotide polymorphisms (SNPs) extracted from patients is 17,891,936. The datasets are available for download from either the publicly accessible GWAS catalog website (https://www.ebi.ac.uk/gwas/downloads/summary-statistics) or the International Epidemiology Unit GWAS database (https://gwas.mrcieu.ac.uk/). All participants in the PD studies have European ancestry.

### 2.2. Data sources for outcome

For individuals aged ≥ 50 years, BMD measurements commonly focus on the forearm, femoral neck, and lumbar spine, which are critical skeletal regions in both postmenopausal women and men.^[17]^ Genetic association study summary data for BMD were employed to assess the impact of PD on overall BMD.^[18]^ Summary statistics for BMD, measured in grams per square centimeter (g/cm²), were obtained by downloading data from the Genetic Factors for Osteoporosis Consortium (GEFOS) website (GEFOS, http://www.gefos.org/). Additionally, GWAS summary statistics for BMD were available from the publicly accessible GWAS catalog website (https://www.ebi.ac.uk/gwas/downloads/summary-statistics) or the International Epidemiology Unit GWAS database (https://gwas.mrcieu.ac.uk/datasets/). This constitutes the largest GWAS study conducted on BMD measured using dual-energy X-ray absorptiometry to date.^[19]^

The forearm BMD (FA-BMD) dataset includes 8143 patients diagnosed with osteoporosis from European women and men, with 9,955,366 SNPs extracted. Similarly, the femoral neck BMD (FN-BMD) dataset comprises 32,735 patients diagnosed with osteoporosis from European women and men, with 10,586,900 SNPs extracted. Furthermore, the lumbar Spine BMD (LS-BMD) dataset encompasses 28,498 patients diagnosed with osteoporosis from European women and men, with 10,582,867 SNPs extracted. All participants in the bone density studies have European ancestry.

### 2.3. Instrumental variable

The selected instrumental variables underwent rigorous scrutiny to ensure adherence to the core assumptions of MR analysis. Relevance was assessed to confirm a robust correlation between the SNPs and the exposure factors, while independence ensured that SNPs remained unaffected by potential confounding factors. Moreover, exclusivity was evaluated to ensure that SNPs influenced the outcome solely through the exposure factors. PhenoScanner was employed to validate any potential confounding associations between the selected instrumental variables and the SNPs.

In our investigation, we conducted an MR analysis to explore the potential causal link between PD and the risk of BMD and its subtypes. To ensure a precise evaluation, specific criteria were defined for the selection of instrumental variables. These included a significance threshold of *P* < 5 × 10^−6^ and linkage disequilibrium with an *r*² < 0.001, utilizing a window size of 10,000 kb. Additionally, SNPs possessing palindromic sequences were excluded from the analysis. Ultimately, this meticulous process enabled us to identify reliable instrumental variables that served as proxies for the subsequent proteins.

### 2.4. Statistical analysis

MR analysis employs genetic variation as an instrumental variable to estimate the causal impact of exposure variables on outcomes.^[20]^ To enhance the robustness of our MR analysis, we performed various sensitivity analyses, including simple mode, weighted mode, inverse variance weighted (IVW), and weighted median.

In this study, our primary methods for estimating the causal relationship between PD and BMD (including FN-BMD, LS-BMD, and FA-BMD) were the IVW method and MR Egger regression. We relied on the IVW results as our main source of information, as these unbiased findings were predicated on the assumption of no horizontal pleiotropy.^[21]^ Moreover, the intercept term in the MR Egger regression provided valuable insights into whether directional horizontal pleiotropy was influencing the MR analysis outcomes.^[22]^ To further corroborate the IVW results, we employed weighted median analyses, which offer more robust estimates across a wide range of scenarios, despite their relatively lower efficiency.^[23]^

Within the context of the MR-PRESSO analysis, our aim was to minimize variability in the estimated causal effect by excluding SNPs that disproportionately contributed to this variability.^[24]^ This analysis entailed 1000 distributions, and heterogeneity was discerned through the IVW method and MR Egger regression. We assessed the presence of heterogeneity using the Cochran *Q* statistic, considering heterogeneity significant if the *P*-value was < .05. Furthermore, to identify potentially influential SNPs, we conducted a “leave-one-out” sensitivity analysis.

All statistical analyses were performed using the Two-Sample MR package in R statistical software version 4.2.3 (R Foundation for Statistical Computing, Vienna, Austria).^[25]^ Significance was defined at *P*-values < .05. To account for multiple testing, we applied a Bonferroni correction, considering associations with Bonferroni-corrected *P*-values < .016 (where *P* = .05/3) as strong evidence of causal associations. Moreover, associations with *P*-values below .05 but above .016 were considered suggestive evidence of associations in the MR analysis. To avoid weak instrument bias (F < 10) in the two-sample model, we estimated the exposure strength of the instrumental variable using the approximation of the F statistic. The calculation of the F value and *R*^2^ adhered to the formula utilized in previous studies.^[25,26]^

### 2.5. Ethics

The data used in this study were obtained from a publicly accessible and shared database, eliminating the requirement for ethical approval or participant consent.

## 3. Results

The comprehensive details pertaining to the studies and datasets are presented in Tables 1 to 5. All participants belonged to the European ethnic group (100%), effectively addressing the concern of ethnic disparities.

**Table 1:** Genetic association study summary data for exposure and outcome.

Exposure/outcome	Sample size	Web source	First author	Consortium	Year	Population
PD	482,730	https://gwas.mrcieu.ac.uk/datasets/	Nalls MA	IPDGC	2019	European
FA-BMD	8143	https://gwas.mrcieu.ac.uk/datasets/	Zheng	GEFOS	2015	European
FN-BMD	32,735	https://gwas.mrcieu.ac.uk/datasets/	Zheng	GEFOS	2015	European
LS-BMD	28,498	https://gwas.mrcieu.ac.uk/datasets/	Zheng	GEFOS	2015	European

FA-BMD = forearm bone mineral, FN-BMD = femoral neck bone mineral density, GEFOS = Genetic Factors for Osteoporosis Consortium, IPDGC = International Parkinson Disease Genomics Consortium, LS-BMD = lumbar spine bone; mineral density, PD = Parkinson disease.

**Table 2:** Single-nucleotide polymorphisms (SNPs) related to constipation at genome-wide significance.

SNP	EA	OA	EAF	Beta	SE	*P*-value	*R* ^2^	F
rs823106	C	G	0.8488	-0.1492	0.0239	4.10E-10	8.07E-05	38.97086
rs142660239	T	G	0.0191	-0.3772	0.0779	1.28E-06	0.0008459	23.44429
rs35749011	A	G	0.0191	0.7508	0.0659	5.02E-30	0.0002688	129.8004
rs114797774	T	C	0.0182	0.3901	0.0796	9.43E-07	4.98E-05	24.01729
rs111972941	G	A	0.0472	0.2482	0.0514	1.37E-06	4.97E-05	23.31715
rs61835654	C	T	0.23	0.1032	0.0216	1.85E-06	4.73E-05	22.82707
rs112413063	C	T	0.0637	-0.1813	0.0396	4.62E-06	4.34E-05	20.96059
rs6715875	C	T	0.0362	0.2902	0.0599	1.25E-06	5.01E-05	23.47142
rs4613239	G	C	0.1326	0.1784	0.0248	6.21E-13	0.0001072	51.74692
rs6741007	G	T	0.4507	-0.1233	0.0175	2.09E-12	0.0001028	49.64188
rs4851487	T	C	0.3907	0.0808	0.0174	3.25E-06	4.47E-05	21.56366
rs73032517	G	A	0.0295	0.2706	0.0558	1.27E-06	4.87E-05	23.51719
rs10513789	G	T	0.1826	-0.1596	0.0219	3.18E-13	0.00011	53.10993
rs9840232	T	C	0.1766	0.1157	0.0233	6.56E-07	5.11E-05	24.65773
rs9845968	G	A	0.5157	0.0842	0.0175	1.42E-06	4.80E-05	23.14975
rs6808178	C	T	0.6228	-0.0864	0.0174	7.20E-07	5.11E-05	24.65626
rs4488803	A	G	0.3746	-0.1136	0.0199	1.08E-08	6.75E-05	32.58732
rs7695720	C	A	0.2091	-0.1255	0.0208	1.53E-09	7.54E-05	36.40483
rs34311866	C	T	0.1958	0.2272	0.0231	7.97E-23	0.0002004	96.73662
rs4698412	A	G	0.553	0.1258	0.0168	7.05E-14	0.0001161	56.07134
rs356203	T	C	0.6169	-0.2398	0.0178	3.01E-41	0.0003758	181.4916
rs4836108	A	G	0.4795	0.1094	0.0225	1.20E-06	5.04E-05	23.6411
rs75646569	G	T	0.1117	0.1916	0.0266	5.62E-13	0.0001075	51.8831
rs41286192	G	A	0.0308	0.3164	0.067	2.30E-06	4.75E-05	22.30085
rs35265698	G	C	0.1547	-0.2	0.0303	3.93E-11	9.06E-05	43.56853
rs2949760	A	G	0.3649	0.0915	0.0193	2.00E-06	4.66E-05	22.47635
rs182621729	T	C	0.0289	0.3083	0.0633	1.11E-06	4.94E-05	23.72126
rs858295	G	A	0.3947	-0.1039	0.0176	3.83E-09	7.22E-05	34.85009
rs7818035	A	G	0.0126	-0.693	0.1429	1.24E-06	0.0017193	23.51464
rs620490	G	T	0.2762	-0.1174	0.019	6.46E-10	7.91E-05	38.17923
rs2208485	A	G	0.415	-0.0936	0.0182	2.65E-07	5.48E-05	26.44887
rs10756905	T	C	0.2409	0.1011	0.0196	2.46E-07	5.51E-05	26.60654
rs144814361	T	C	0.0174	0.4411	0.068	9.07E-11	8.72E-05	42.07794
rs329647	C	G	0.6662	-0.1133	0.0178	1.94E-10	8.39E-05	40.5152
rs10766301	T	C	0.4104	0.0907	0.0192	2.21E-06	4.62E-05	22.31569
rs10847864	T	G	0.3625	0.1274	0.0179	9.81E-13	0.0001049	50.65601
rs75505347	T	C	0.0195	0.3917	0.0674	6.12E-09	7.00E-05	33.77424
rs79436216	G	A	0.0351	0.2816	0.0603	3.02E-06	4.65E-05	21.80865
rs28370649	G	A	0.0272	0.2836	0.0547	2.20E-07	5.57E-05	26.88042
rs4774417	A	G	0.7397	0.1052	0.0192	4.63E-08	6.22E-05	30.02114
rs34679758	G	A	0.1216	-0.1275	0.0259	8.72E-07	5.02E-05	24.23366
rs12934900	T	A	0.6571	0.1215	0.0184	4.33E-11	9.03E-05	43.60287
rs12929797	T	C	0.4285	0.082	0.017	1.32E-06	4.82E-05	23.26634
rs847685	C	G	0.2028	-0.1483	0.0293	3.98E-07	5.47E-05	25.618
rs10451230	T	A	0.565	-0.096	0.0175	4.42E-08	6.23E-05	30.09294
rs58879558	C	T	0.2229	-0.2383	0.025	1.36E-21	0.0001882	90.85865
rs4588066	A	G	0.326	0.1046	0.0178	4.45E-09	7.15E-05	34.53199
rs145217940	A	T	0.0199	0.4186	0.0852	8.91E-07	5.15E-05	24.1389
rs2295547	C	A	0.32	-0.0894	0.0182	8.77E-07	5.00E-05	24.12851
rs4810687	T	G	0.5642	0.0932	0.0187	6.27E-07	5.15E-05	24.83973
rs2248244	A	G	0.2898	0.1001	0.0211	2.00E-06	4.66E-05	22.50616

EA = effect allele, EAF = effect allele frequency, OA = other allele, SE = standard error, SNP = single-nucleotide polymorphism.

**Table 3:** Details of the IVs used for MR analysis [causal effect of PD on FN-BMD].

SNP	EA	OA	Exposure				Outcome				*R* ^2^	F
			Beta	EAF	SE	*P*-value	Beta	EAF	SE	*P*-value		
rs10451230	T	A	-0.096	0.565	0.0175	4.42E-08	-0.0104	0.5339	0.0076	.1788	6.23E-05	30.09294
rs10513789	G	T	-0.1596	0.1826	0.0219	3.18E-13	-0.0056	0.242	0.0095	.5651	0.000110008	53.10993
rs10756905	T	C	0.1011	0.2409	0.0196	2.46E-07	-0.006	0.2243	0.0089	.5084	5.51E-05	26.60654
rs10766301	T	C	0.0907	0.4104	0.0192	2.21E-06	0.01322	0.356	0.0076	.0899	4.62E-05	22.31569
rs10847864	T	G	0.1274	0.3625	0.0179	9.81E-13	0.00942	0.3404	0.0087	.2924	0.000104926	50.65601
rs111972941	G	A	0.2482	0.0472	0.0514	1.37E-06	0.01089	0.0323	0.0185	.5649	4.97E-05	23.31715
rs112413063	C	T	-0.1813	0.0637	0.0396	4.62E-06	0.00641	0.0761	0.0155	.6853	4.34E-05	20.96059
rs114797774	T	C	0.3901	0.0182	0.0796	9.43E-07	0.09755	0.0158	0.0319	.0027	4.98E-05	24.01729
rs12929797	T	C	0.082	0.4285	0.017	1.32E-06	-0.009	0.3932	0.0086	.3078	4.82E-05	23.26634
rs12934900	T	A	0.1215	0.6571	0.0184	4.33E-11	0.00623	0.5677	0.0079	.4418	9.03E-05	43.60287
rs145217940	A	T	0.4186	0.0199	0.0852	8.91E-07	-0.0216	0.0166	0.0295	.4753	5.15E-05	24.1389
rs182621729	T	C	0.3083	0.0289	0.0633	1.11E-06	0.04126	0.0269	0.0249	.1043	4.94E-05	23.72126
rs2208485	A	G	-0.0936	0.415	0.0182	2.65E-07	-0.0044	0.4098	0.0077	.5794	5.48E-05	26.44887
rs2248244	A	G	0.1001	0.2898	0.0211	2.00E-06	0.00831	0.2951	0.0085	.337	4.66E-05	22.50616
rs2295547	C	A	-0.0894	0.32	0.0182	8.77E-07	-0.0004	0.3275	0.008	.9628	5.00E-05	24.12851
rs28370649	G	A	0.2836	0.0272	0.0547	2.20E-07	0.04485	0.0154	0.0272	.1066	5.57E-05	26.88042
rs2949760	A	G	0.0915	0.3649	0.0193	2.00E-06	0.00535	0.3441	0.0078	.501	4.66E-05	22.47635
rs329647	C	G	-0.1133	0.6662	0.0178	1.94E-10	0.0084	0.6629	0.0081	.3092	8.39E-05	40.5152
rs34679758	G	A	-0.1275	0.1216	0.0259	8.72E-07	-0.0034	0.0954	0.0114	.7673	5.02E-05	24.23366
rs35265698	G	C	-0.2	0.1547	0.0303	3.93E-11	-0.0201	0.2459	0.012	.1014	9.06E-05	43.56853
rs356203	T	C	-0.2398	0.6169	0.0178	3.01E-41	-0.0066	0.5795	0.008	.4218	0.000375829	181.4916
rs35749011	A	G	0.7508	0.0191	0.0659	5.02E-30	-0.0031	0.0111	0.0325	.9251	0.000268817	129.8004
rs4488803	A	G	-0.1136	0.3746	0.0199	1.08E-08	-0.001	0.4589	0.0078	.8977	6.75E-05	32.58732
rs4588066	A	G	0.1046	0.326	0.0178	4.45E-09	-0.0094	0.3247	0.0081	.2543	7.15E-05	34.53199
rs4613239	G	C	0.1784	0.1326	0.0248	6.21E-13	0.01295	0.154	0.0114	.2647	0.000107185	51.74692
rs4698412	A	G	0.1258	0.553	0.0168	7.05E-14	-0.0059	0.4924	0.0075	.4412	0.000116142	56.07134
rs4774417	A	G	0.1052	0.7397	0.0192	4.63E-08	0.00671	0.712	0.0085	.4371	6.22E-05	30.02114
rs4810687	T	G	0.0932	0.5642	0.0187	6.27E-07	-0.0019	0.6343	0.0076	.8025	5.15E-05	24.83973
rs4836108	A	G	0.1094	0.4795	0.0225	1.20E-06	-0.0041	0.5049	0.0076	.601	5.04E-05	23.6411
rs4851487	T	C	0.0808	0.3907	0.0174	3.25E-06	-0.0026	0.4446	0.0077	.7372	4.47E-05	21.56366
rs58879558	C	T	-0.2383	0.2229	0.025	1.36E-21	-0.0263	0.2144	0.0109	.0187	0.000188184	90.85865
rs61835654	C	T	0.1032	0.23	0.0216	1.85E-06	-0.0076	0.2381	0.0094	.4239	4.73E-05	22.82707
rs620490	G	T	-0.1174	0.2762	0.019	6.46E-10	-0.0008	0.3235	0.0082	.9273	7.91E-05	38.17923
rs6715875	C	T	0.2902	0.0362	0.0599	1.25E-06	0.02128	0.0419	0.0213	.3278	5.01E-05	23.47142
rs6741007	G	T	-0.1233	0.4507	0.0175	2.09E-12	-0.004	0.5607	0.009	.6622	0.000102826	49.64188
rs6808178	C	T	-0.0864	0.6228	0.0174	7.20E-07	0.00067	0.6776	0.0077	.9322	5.11E-05	24.65626
rs73032517	G	A	0.2706	0.0295	0.0558	1.27E-06	0.00896	0.0186	0.0236	.7102	4.87E-05	23.51719
rs75505347	T	C	0.3917	0.0195	0.0674	6.12E-09	0.02592	0.0112	0.0334	.4477	7.00E-05	33.77424
rs75646569	G	T	0.1916	0.1117	0.0266	5.62E-13	-0.0064	0.1091	0.0128	.6264	0.000107467	51.8831
rs7695720	C	A	-0.1255	0.2091	0.0208	1.53E-09	0.0013	0.2133	0.0092	.8905	7.54E-05	36.40483
rs7818035	A	G	-0.693	0.0126	0.1429	1.24E-06	0.04669	0.0155	0.0429	.2864	0.001719345	23.51464
rs79436216	G	A	0.2816	0.0351	0.0603	3.02E-06	-0.0037	0.0239	0.0206	.8594	4.65E-05	21.80865
rs823106	C	G	-0.1492	0.8488	0.0239	4.10E-10	0.00629	0.7862	0.011	.5756	8.07E-05	38.97086
rs847685	C	G	-0.1483	0.2028	0.0293	3.98E-07	0.01951	0.2064	0.0096	.0472	5.47E-05	25.618
rs858295	G	A	-0.1039	0.3947	0.0176	3.83E-09	-0.0087	0.4033	0.0077	.2719	7.22E-05	34.85009
rs9840232	T	C	0.1157	0.1766	0.0233	6.56E-07	0.01974	0.1284	0.01	.0524	5.11E-05	24.65773
rs9845968	G	A	0.0842	0.5157	0.0175	1.42E-06	0.01514	0.609	0.0075	.0483	4.80E-05	23.14975

EA = effect allele, EAF = effect allele frequency, OA = other allele, SE = standard error, SNP = single-nucleotide polymorphism.

**Table 4:** Details of the IVs used for MR analysis [causal effect of PD on FB-BMD].

SNP	EA	OA	Beta	Exposure			Beta	Outcome			*R* ^2^	F
				EAF	SE	*P*-value		EAF	SE	*P*-value		
rs10451230	T	A	-0.096	0.565	0.0175	4.42E-08	-0.038	0.5339	0.0161	.02041	6.23E-05	30.09294
rs10513789	G	T	-0.16	0.1826	0.0219	3.18E-13	-0.002	0.242	0.0197	.91872	0.00011001	53.10993
rs10756905	T	C	0.1011	0.2409	0.0196	2.46E-07	0.0234	0.2243	0.0184	.21363	5.51E-05	26.60654
rs10766301	T	C	0.0907	0.4104	0.0192	2.21E-06	0.0257	0.356	0.0157	.10845	4.62E-05	22.31569
rs10847864	T	G	0.1274	0.3625	0.0179	9.81E-13	-0.002	0.3404	0.0172	.90557	0.00010493	50.65601
rs111972941	G	A	0.2482	0.0472	0.0514	1.37E-06	-0.017	0.0323	0.0386	.65925	4.97E-05	23.31715
rs112413063	C	T	-0.181	0.0637	0.0396	4.62E-06	0.0239	0.0761	0.0324	.46915	4.34E-05	20.96059
rs114797774	T	C	0.3901	0.0182	0.0796	9.43E-07	0.1068	0.0158	0.0591	.07645	4.98E-05	24.01729
rs12929797	T	C	0.082	0.4285	0.017	1.32E-06	-0.006	0.3932	0.0207	.76472	4.82E-05	23.26634
rs12934900	T	A	0.1215	0.6571	0.0184	4.33E-11	-0.01	0.5677	0.0168	.56666	9.03E-05	43.60287
rs142660239	T	G	-0.377	0.0191	0.0779	1.28E-06	-0.042	0.0095	0.0659	.52905	0.00084592	23.44429
rs145217940	A	T	0.4186	0.0199	0.0852	8.91E-07	0.0561	0.0166	0.055	.31762	5.15E-05	24.1389
rs182621729	T	C	0.3083	0.0289	0.0633	1.11E-06	-0.03	0.0269	0.0505	.56054	4.94E-05	23.72126
rs2208485	A	G	-0.094	0.415	0.0182	2.65E-07	-0.003	0.4098	0.0157	.84311	5.48E-05	26.44887
rs2248244	A	G	0.1001	0.2898	0.0211	2.00E-06	0.0083	0.2951	0.0175	.63958	4.66E-05	22.50616
rs2295547	C	A	-0.089	0.32	0.0182	8.77E-07	0.0039	0.3281	0.0165	.81908	5.00E-05	24.12851
rs28370649	G	A	0.2836	0.0272	0.0547	2.20E-07	-0.006	0.0154	0.0557	.92198	5.57E-05	26.88042
rs2949760	A	G	0.0915	0.3649	0.0193	2.00E-06	0.0246	0.3441	0.016	.1322	4.66E-05	22.47635
rs329647	C	G	-0.113	0.6662	0.0178	1.94E-10	-0.006	0.6629	0.0169	.7084	8.39E-05	40.5152
rs34679758	G	A	-0.128	0.1216	0.0259	8.72E-07	0.0091	0.0954	0.0234	.70349	5.02E-05	24.23366
rs35265698	G	C	-0.2	0.1547	0.0303	3.93E-11	-0.011	0.2459	0.0246	.66809	9.06E-05	43.56853
rs356203	T	C	-0.24	0.6169	0.0178	3.01E-41	0.0135	0.5795	0.0159	.40415	0.00037583	181.4916
rs35749011	A	G	0.7508	0.0191	0.0659	5.02E-30	-0.004	0.0111	0.0633	.94834	0.00026882	129.8004
rs4488803	A	G	-0.114	0.3746	0.0199	1.08E-08	0.0007	0.4589	0.0163	.96656	6.75E-05	32.58732
rs4588066	A	G	0.1046	0.326	0.0178	4.45E-09	-0.004	0.3247	0.0168	.82816	7.15E-05	34.53199
rs4613239	G	C	0.1784	0.1326	0.0248	6.21E-13	-0.013	0.154	0.0234	.58648	0.00010719	51.74692
rs4698412	A	G	0.1258	0.553	0.0168	7.05E-14	0.0017	0.4924	0.0154	.91468	0.00011614	56.07134
rs4774417	A	G	0.1052	0.7397	0.0192	4.63E-08	-0.02	0.712	0.0174	.25653	6.22E-05	30.02114
rs4810687	T	G	0.0932	0.5642	0.0187	6.27E-07	0.0091	0.6343	0.0157	.5715	5.15E-05	24.83973
rs4836108	A	G	0.1094	0.4795	0.0225	1.20E-06	0.0041	0.5049	0.0155	.79689	5.04E-05	23.6411
rs4851487	T	C	0.0808	0.3907	0.0174	3.25E-06	-0.011	0.4446	0.0159	.50629	4.47E-05	21.56366
rs58879558	C	T	-0.238	0.2229	0.025	1.36E-21	-0.041	0.2144	0.0327	.21778	0.00018818	90.85865
rs61835654	C	T	0.1032	0.23	0.0216	1.85E-06	-0.003	0.2381	0.0194	.87838	4.73E-05	22.82707
rs620490	G	T	-0.117	0.2762	0.019	6.46E-10	-0.015	0.3235	0.0172	.39003	7.91E-05	38.17923
rs6715875	C	T	0.2902	0.0362	0.0599	1.25E-06	-0.012	0.0419	0.0416	.77131	5.01E-05	23.47142
rs6741007	G	T	-0.123	0.4507	0.0175	2.09E-12	-0.027	0.5607	0.0213	.21096	0.00010283	49.64188
rs6808178	C	T	-0.086	0.6228	0.0174	7.20E-07	-0.023	0.6776	0.0159	.16285	5.11E-05	24.65626
rs73032517	G	A	0.2706	0.0295	0.0558	1.27E-06	0.0078	0.0186	0.047	.87013	4.87E-05	23.51719
rs75505347	T	C	0.3917	0.0195	0.0674	6.12E-09	0.0717	0.0112	0.0616	.25316	7.00E-05	33.77424
rs75646569	G	T	0.1916	0.1117	0.0266	5.62E-13	0.0136	0.1091	0.0269	.62109	0.00010747	51.8831
rs7695720	C	A	-0.126	0.2091	0.0208	1.53E-09	0.0021	0.2133	0.0192	.91474	7.54E-05	36.40483
rs7818035	A	G	-0.693	0.0126	0.1429	1.24E-06	-0.099	0.0155	0.081	.23017	0.00171935	23.51464
rs79436216	G	A	0.2816	0.0351	0.0603	3.02E-06	0.0714	0.0239	0.0419	.09503	4.65E-05	21.80865
rs823106	C	G	-0.149	0.8488	0.0239	4.10E-10	-0.037	0.7862	0.0233	.11639	8.07E-05	38.97086
rs847685	C	G	-0.148	0.2028	0.0293	3.98E-07	-0.019	0.2064	0.0192	.33021	5.47E-05	25.618
rs858295	G	A	-0.104	0.3947	0.0176	3.83E-09	-0.008	0.4033	0.0164	.63686	7.22E-05	34.85009
rs9840232	T	C	0.1157	0.1766	0.0233	6.56E-07	0.0064	0.1284	0.0207	.76156	5.11E-05	24.65773
rs9845968	G	A	0.0842	0.5157	0.0175	1.42E-06	-0.013	0.609	0.0156	.40001	4.80E-05	23.14975

EA = effect allele, EAF = effect allele frequency, OA = other allele, SE = standard error, SNP = single-nucleotide polymorphism.

**Table 5:** Details of the IVs used for MR analysis [causal effect of PD on LS-BMD].

SNP	EA	OA	Beta	Exposure			Beta	Outcome			*R* ^2^	F
				EAF	SE	*P*-value		EAF	SE	*P*-value		
rs10451230	T	A	-0.096	0.565	0.0175	4.42E-08	-0.02	0.5339	0.0089	.0251	6.23E-05	30.09294
rs10513789	G	T	-0.16	0.1826	0.0219	3.18E-13	-0.017	0.242	0.011	.1359	0.00011	53.10993
rs10756905	T	C	0.1011	0.2409	0.0196	2.46E-07	-0.012	0.2243	0.0104	.2554	5.51E-05	26.60654
rs10766301	T	C	0.0907	0.4104	0.0192	2.21E-06	-0.004	0.356	0.0089	.6581	4.62E-05	22.31569
rs10847864	T	G	0.1274	0.3625	0.0179	9.81E-13	0.0238	0.3404	0.0102	.022	0.0001	50.65601
rs111972941	G	A	0.2482	0.0472	0.0514	1.37E-06	-0.001	0.0323	0.0216	.9515	4.97E-05	23.31715
rs112413063	C	T	-0.181	0.0637	0.0396	4.62E-06	0.0103	0.0761	0.0181	.5775	4.34E-05	20.96059
rs114797774	T	C	0.3901	0.0182	0.0796	9.43E-07	0.0659	0.0158	0.0378	.0891	4.98E-05	24.01729
rs12929797	T	C	0.082	0.4285	0.017	1.32E-06	-0.006	0.3932	0.0105	.6062	4.82E-05	23.26634
rs12934900	T	A	0.1215	0.6571	0.0184	4.33E-11	-0.005	0.5677	0.0093	.6064	9.03E-05	43.60287
rs145217940	A	T	0.4186	0.0199	0.0852	8.91E-07	0.0118	0.0166	0.0344	.7366	5.15E-05	24.1389
rs182621729	T	C	0.3083	0.0289	0.0633	1.11E-06	0.0274	0.0269	0.0284	.3472	4.94E-05	23.72126
rs2208485	A	G	-0.094	0.415	0.0182	2.65E-07	-0.003	0.4098	0.009	.7674	5.48E-05	26.44887
rs2248244	A	G	0.1001	0.2898	0.0211	2.00E-06	0.0048	0.2951	0.0099	.6378	4.66E-05	22.50616
rs2295547	C	A	-0.089	0.32	0.0182	8.77E-07	0.0041	0.3275	0.0092	.6622	5.00E-05	24.12851
rs28370649	G	A	0.2836	0.0272	0.0547	2.20E-07	0.0375	0.0154	0.031	.2368	5.57E-05	26.88042
rs2949760	A	G	0.0915	0.3649	0.0193	2.00E-06	-0.005	0.3441	0.0091	.5566	4.66E-05	22.47635
rs329647	C	G	-0.113	0.6662	0.0178	1.94E-10	0.0034	0.6629	0.0094	.7243	8.39E-05	40.5152
rs34679758	G	A	-0.128	0.1216	0.0259	8.72E-07	0.0021	0.0954	0.0133	.8745	5.02E-05	24.23366
rs35265698	G	C	-0.2	0.1547	0.0303	3.93E-11	-0.012	0.2459	0.0132	.372	9.06E-05	43.56853
rs356203	T	C	-0.24	0.6169	0.0178	3.01E-41	-0.012	0.5795	0.009	.2066	0.00038	181.4916
rs35749011	A	G	0.7508	0.0191	0.0659	5.02E-30	0.015	0.0111	0.0367	.6899	0.00027	129.8004
rs4488803	A	G	-0.114	0.3746	0.0199	1.08E-08	-0.003	0.4589	0.009	.7497	6.75E-05	32.58732
rs4588066	A	G	0.1046	0.326	0.0178	4.45E-09	-0.007	0.3247	0.0094	.4549	7.15E-05	34.53199
rs4613239	G	C	0.1784	0.1326	0.0248	6.21E-13	0.0185	0.154	0.0133	.1738	0.00011	51.74692
rs4698412	A	G	0.1258	0.553	0.0168	7.05E-14	-0.01	0.4924	0.0087	.2618	0.00012	56.07134
rs4774417	A	G	0.1052	0.7397	0.0192	4.63E-08	0.0028	0.712	0.0098	.7833	6.22E-05	30.02114
rs4810687	T	G	0.0932	0.5642	0.0187	6.27E-07	-0.019	0.6343	0.0088	.0365	5.15E-05	24.83973
rs4836108	A	G	0.1094	0.4795	0.0225	1.20E-06	-0.006	0.5049	0.0088	.5191	5.04E-05	23.6411
rs4851487	T	C	0.0808	0.3907	0.0174	3.25E-06	-0.015	0.4446	0.009	.1086	4.47E-05	21.56366
rs58879558	C	T	-0.238	0.2229	0.025	1.36E-21	-0.053	0.2144	0.0138	.0002	0.00019	90.85865
rs61835654	C	T	0.1032	0.23	0.0216	1.85E-06	0.0056	0.2381	0.0109	.6142	4.73E-05	22.82707
rs620490	G	T	-0.117	0.2762	0.019	6.46E-10	0.0001	0.3235	0.0096	.9907	7.91E-05	38.17923
rs6715875	C	T	0.2902	0.0362	0.0599	1.25E-06	0.0481	0.0419	0.0249	.0588	5.01E-05	23.47142
rs6741007	G	T	-0.123	0.4507	0.0175	2.09E-12	0.0082	0.5607	0.011	.4662	0.0001	49.64188
rs6808178	C	T	-0.086	0.6228	0.0174	7.20E-07	-0.008	0.6776	0.009	.3633	5.11E-05	24.65626
rs73032517	G	A	0.2706	0.0295	0.0558	1.27E-06	-0.012	0.0186	0.0271	.665	4.87E-05	23.51719
rs75505347	T	C	0.3917	0.0195	0.0674	6.12E-09	-0.007	0.0112	0.0385	.8537	7.00E-05	33.77424
rs75646569	G	T	0.1916	0.1117	0.0266	5.62E-13	-0.01	0.1091	0.015	.517	0.00011	51.8831
rs7695720	C	A	-0.126	0.2091	0.0208	1.53E-09	-0.019	0.2133	0.0107	.0842	7.54E-05	36.40483
rs7818035	A	G	-0.693	0.0126	0.1429	1.24E-06	-0.002	0.0155	0.0494	.9655	0.00172	23.51464
rs79436216	G	A	0.2816	0.0351	0.0603	3.02E-06	-0.013	0.0239	0.0238	.5823	4.65E-05	21.80865
rs823106	C	G	-0.149	0.8488	0.0239	4.10E-10	0.0022	0.7862	0.0127	.8651	8.07E-05	38.97086
rs847685	C	G	-0.148	0.2028	0.0293	3.98E-07	0.0132	0.2064	0.0111	.2457	5.47E-05	25.618
rs858295	G	A	-0.104	0.3947	0.0176	3.83E-09	-0.017	0.4033	0.009	.0657	7.22E-05	34.85009
rs9840232	T	C	0.1157	0.1766	0.0233	6.56E-07	-0.013	0.1284	0.0116	.2804	5.11E-05	24.65773
rs9845968	G	A	0.0842	0.5157	0.0175	1.42E-06	0.0173	0.609	0.0087	.0536	4.80E-05	23.14975

EA = effect allele, EAF = effect allele frequency, OA = other allele, SE = standard error, SNP = single-nucleotide polymorphism.

### 3.1. Association between bone mineral density and genetic variants

**Figure 2.**
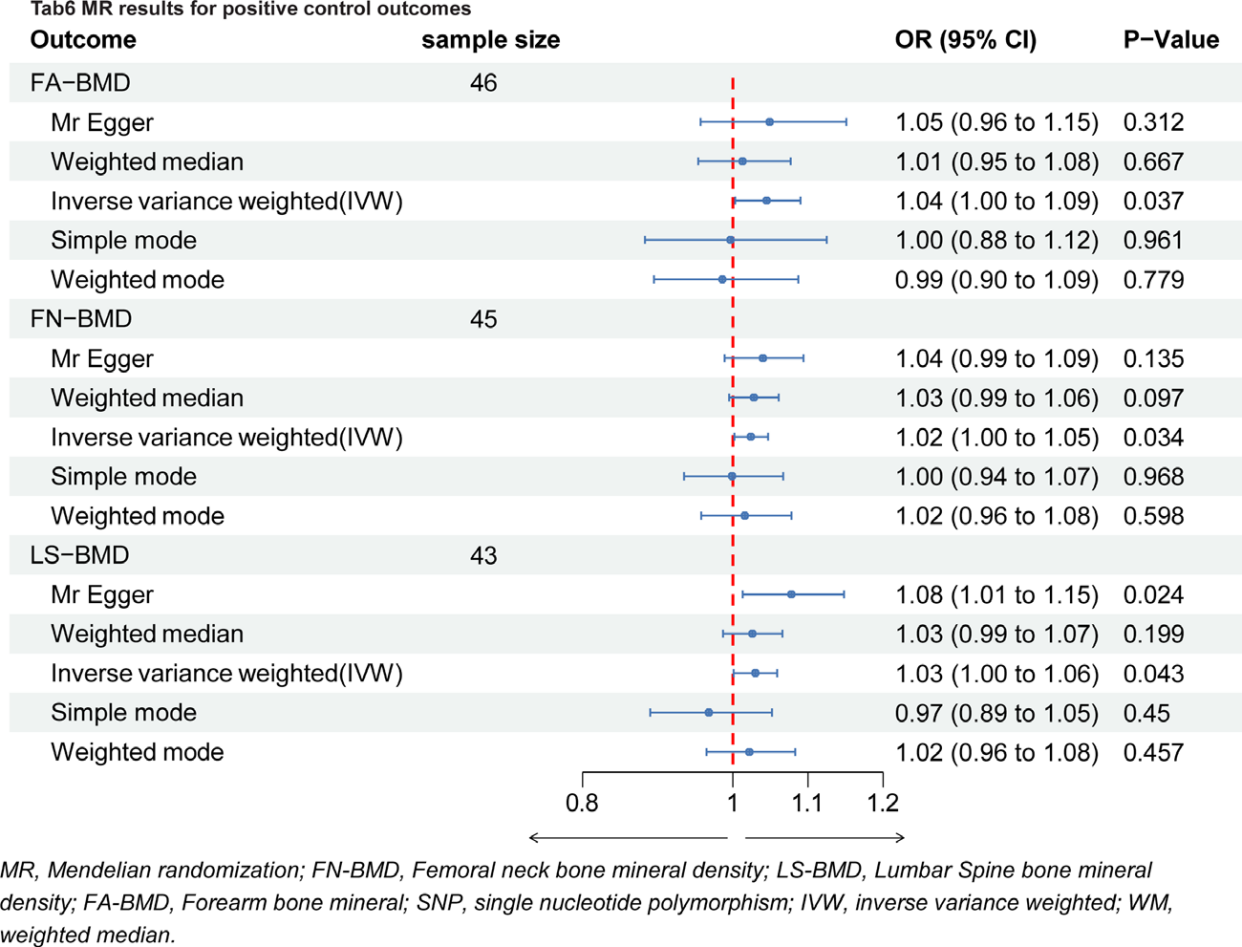
MR results for positive control outcomes. MR = Mendelian randomization; FN-BMD = femoral neck bone mineral density; LS-BMD = lumbar spine bone mineral density; FA-BMD = forearm bone mineral; SNP = single-nucleotide polymorphism; IVW = inverse variance weighted; WM = weighted median.

**Figure 3.**
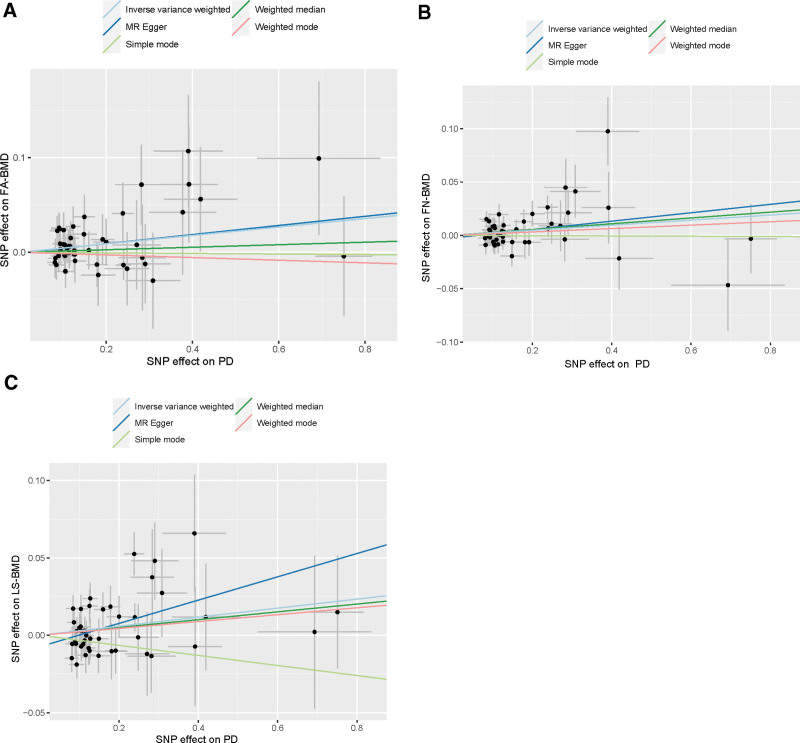
Scatter plots of the Mendelian randomization analyses for the association of TB-BMD with the risk of hip OA. The red line represents the inverse variance weighted estimate, the green line represents the weighted median estimate, the blue line represents the MR Egger estimate. BMD = bone mineral density; OA = other allele.

The data were subjected to comprehensive statistical analysis to explore the relationships between different measures of BMD and genetic variants. We employed several statistical methods, including MR Egger, weighted median, and IVW, to assess the potential associations. In the context of MR, the IVW method was primarily considered for evaluating the results presented in Figure 2 and depicted in Figure 3.

### 3.2. FA-BMD statistical analysis

For the FA-BMD analysis, the IVW method yielded a *P*-value of .037, indicating a possible significant relationship between specific SNPs and FA-BMD (odds ratio [OR], 1.05; 95% confidence interval [CI], 1.00–1.09). Conversely, the MR Egger method yielded a *P*-value of .312, suggesting a potential nonsignificant association between genetic variants and FA-neck BMD (OR, 1.05; 95% CI, 0.96–1.15). Overall, the analysis suggests potential links between genetic variants and bone density for FA-BMD, although statistical significance was not consistently observed.

### 3.3. FN-BMD statistical analysis

For the analysis of FN-BMD, the IVW method yielded a *P*-value of .034, indicating a possible significant relationship between specific SNPs and FN-BMD (OR, 1.02; 95% CI, 1.00–1.05). Conversely, the MR Egger method yielded a *P*-value of .135, suggesting a potential nonsignificant association between genetic variants and FN-BMD (OR, 1.04; 95% CI, 0.99–1.09). The analysis of FN-BMD indicates potential associations between genetic variants and bone density, with varying levels of significance depending on the statistical method employed.

### 3.4. LS-BMD statistical analysis

Due to the presence of confounding SNPs in LS-BMD analysis, we excluded 2 confounding factors, namely rs10766301 and rs10756905. For the subsequent analysis of LS-BMD, the IVW method yielded a *P*-value of .043, suggesting a possible significant relationship between certain SNPs and LS-BMD (OR, 1.03; 95% CI, 1.00–1.06). The MR Egger method yielded a *P*-value of .024, indicating a potentially significant association between genetic variants and LS-BMD (OR, 1.08; 95% CI, 1.01–1.15). Collectively, the analysis of LS-BMD suggests potential associations between genetic variants and bone density, with varying levels of significance observed across different statistical methods.

### 3.5. Sensitivity analysis

**Figure 4.**
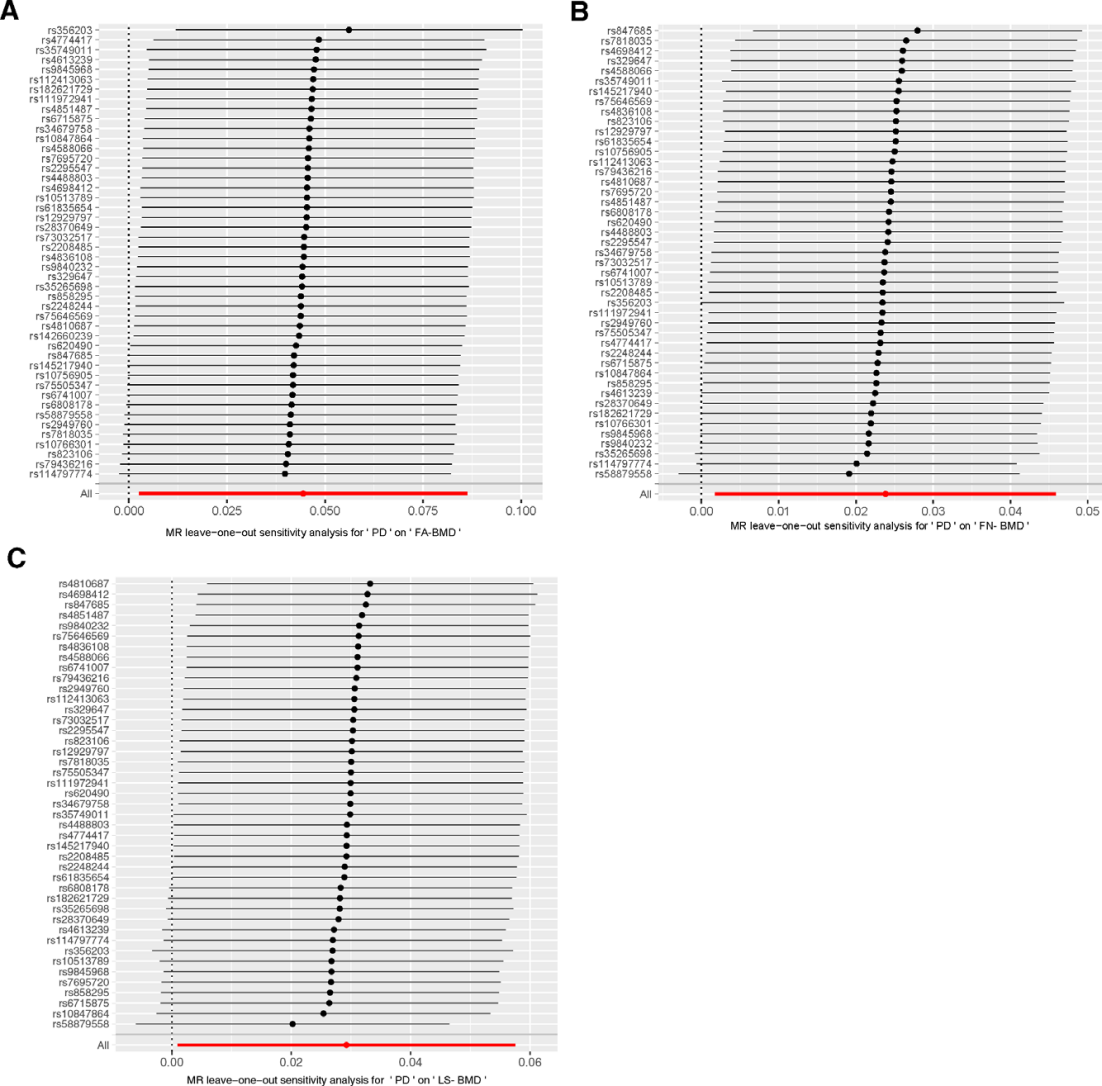
(A) Forrest plot of the causal effects of PD-associated SNPs on hip FA-BMD. (B) Forrest plot of the causal effects of PD-associated SNPs on hip FN-BMD. (C) Forrest plot of the causal effects of PD-associated SNPs on hip LS-BMD. FA-BMD = forearm bone mineral density; FN-BMD = femoral neck bone mineral density; LS-BMD = lumbar spine bone mineral density; SNPs = single-nucleotide polymorphisms.

We conducted sensitivity analyses to explore the associations between bone density measurements and genetic variants. For FA-BMD, the MR-PRESSO method (RSSobs = 36.09; *P* = .928), Cochran Q test (Q = 29.14), and heterogeneity *P*-value (.97) were utilized to detect potential pleiotropy issues. Similarly, for FN-BMD, we employed the MR-PRESSO method (RSSobs = 54.69; *P* = .249), Cochran Q test (Q = 51.15), and heterogeneity *P*-value (.21) to ascertain potential pleiotropy concerns. Additionally, regarding LS-BMD, the MR-PRESSO method (RSSobs = 44.25; *P* = .292), Cochran Q test (Q = 57.95), and heterogeneity *P*-value (.052) were used to explore potential pleiotropy issues. These consistently observed patterns of results indicate that none of these analyses identified significant pleiotropy issues across all bone density measurements. Comprehensive details pertaining to the studies and datasets are presented in Table 6. The “leave-one-out” analysis demonstrated that no individual SNP exerts a definitive influence on causal inference (Fig. 4).

**Table 6:** Results of heterogeneity by the Cochran Q test and the MR-PRESSO global test.

Outcome	MR-PRESSO		Pleio *P*-value	Feor *P*-value	Reor *P*-value	Cochran Q	Heterogeneity *P*-value
	RSSobs	*P*-value					
FA-BMD	36.08845	.928	.929	.037*	.044*	29.140	.968
FN-BMD	54.69425	.249	.507	.022*	.034*	51.146	.214
LS-BMD	44.25757	.292	.116	.017*	.043*	57.954	.051

FA-BMD = forearm bone mineral, FN-BMD = femoral neck bone mineral density, LS-BMD = lumbar spine bone mineral density, MR = Mendelian randomization.

* Statistically significant *P*-value.

## 4. Discussion

In this study, we conducted an MR analysis using 2 independent samples to investigate the risk relationship between PD and BMD metrics (FA-BMD, FN-BMD, and LS-BMD) based on 51 SNPs. This MR approach employed genetic variation to mitigate potential confounders. To estimate the causal relationship between PD and BMD, we used the IVW method and MR Egger regression, complemented by MR-PRESSO analysis to address the issues of pleiotropy and heterogeneity.

The IVW results were used as the primary source of information, assuming no horizontal pleiotropy. The intercept term of MR Egger regression provided a powerful tool for detecting directional horizontal pleiotropy, while MR-PRESSO analysis helped correct for the effects of pleiotropy. In our study, we performed a two-sample MR analysis using data from the International Parkinson Disease Genomics Consortium and the GWAS Statistical Abstract of GEFOS. Our findings revealed that PD was weakly positively correlated with the risk of BMD-related markers, suggesting that as the risk of PD exposure increases, the likelihood of osteoporosis also increases.

In our study, we estimated the causal relationship between PD and BMD (including FA-BMD, FN-BMD, and LS-BMD) using the IVW method and MR Egger regression, combined with MR-PRESSO analysis to address pleiotropy and heterogeneity issues. The selection of instrumental variables strictly adhered to the principles of relevance, independence, and exclusivity, and was screened by a functional annotation database. The MR Egger regression results showed that none of the pleiotropy *P*-values were significant (FA-BMD: 0.928, FN-BMD: 0.249, and LS-BMD: 0.292), suggesting no significant pleiotropy in the instrumental variables overall. The MR-PRESSO method further corrected for pleiotropy effects.

Cochran Q statistic was used to assess heterogeneity among instrumental variables. The results indicated no significant heterogeneity for FA-BMD (Q = 29.140, *P* = .968) and FN-BMD (Q = 51.146, *P*0.214), while LS-BMD (Q = 57.954, *P* = .051) was close to, but did not reach the significance level. This suggests that the effects between instrumental variables are consistent in most cases, and heterogeneity is less of an issue. The “leave-one-out” analysis by sequentially removing each SNP and reanalyzing ensured that individual SNPs did not disproportionately influence the overall results, further validating the robustness of our findings. Combining results from multiple methods, we ensured the robustness and credibility of our conclusions.

Several hypotheses have been proposed to elucidate the potential mechanisms between PD and osteoporosis. One hypothesis suggests that motor impairments and postural instability in patients with PD may lead to frequent falls, thereby increasing the risk of fractures.^[27,28]^ Additionally, bone density in patients with PD may be influenced by the disease itself and its therapeutic medications, both of which can affect the incidence of fractures.^[29]^ Furthermore, PD may affect bone metabolism, further increasing the risk of osteoporosis.^[29–32]^ However, these potential mechanisms require further research for validation.

Epidemiological studies have indicated that the prevalence of PD is associated with an increased risk of fractures, with the severity of PD exhibiting a linear relationship with the fracture risk.^[33]^ While patients with PD have an elevated risk of fractures, particularly hip fractures due to an elevated risk of falling, fractures are also associated with lower BMD, which tends to be reduced in this patient group compared to age- and sex-matched controls.^[31]^ Various factors contribute to the elevated fracture susceptibility in patients with PD, including an increased risk of osteoporosis.^[30]^

The causal relationship between PD and BMD remains unestablished in clinical research.^[10–13,34]^ Unlike observational studies, MR analysis employs specific genetic variants that satisfy the instrumental variable assumption to investigate causal relationships in epidemiological research, minimizing the potential for inherent bias.^[35]^ Our study contributes to this field by providing an MR analysis of the relationship between PD and BMD, revealing both congruencies and disparities when compared to prior studies. While our IVW analysis indicates associations between PD and certain BMD measures, previous research might have reported inconsistent outcomes.

However, our study had some limitations. First, all GWAS data used were from European populations. Although this minimizes bias from population stratification, it may limit the generalizability of our findings to other ethnic groups. Second, PD was weakly and positively correlated with the risk of BMD-related indicators, and the strength of these associations was modest. Third, the SNPs used as genetic tools exhibited weak associations with PD (*P* < 5 × 10^−6^), potentially explaining only a portion of the variation in exposure. This limitation could affect the statistical validity of our causality estimates.

## 5. Conclusions

In conclusion, our study aimed to assess the causal effect of PD on BMD using two-sample MR analysis. Our findings suggest a potential causal effect of genetically predicted PD on FA-BMD, FN-BMD, and LS-BMD. However, it is important to acknowledge the complexity of genetic interactions and potential biases inherent in the analysis. Future research should delve deeper into these intricacies, exploring gene–gene interactions, functional mutations, and gene–environment interplay. Our study underscores the importance of adopting measures and collaborative initiatives aimed at preventing bone loss and initiating early interventions for osteoporosis upon the diagnosis of patients with PD.

## Acknowledgments

We would like to thank Editage (www.editage.cn) for English language editing.

## Author contributions

**Data curation:** Qing Long Li, Bing Feng Mo.

**Funding acquisition:** Hong Mian Li.

**Resources:** Dong Yin.

**Software:** Song Guo.

**Writing – original draft:** Yu Huang.

**Writing – review & editing:** Nan Yi.
